# Artificial neural network model with different backpropagation algorithms and meteorological data for solar radiation prediction

**DOI:** 10.1038/s41598-022-13532-3

**Published:** 2022-06-21

**Authors:** Seah Yi Heng, Wanie M. Ridwan, Pavitra Kumar, Ali Najah Ahmed, Chow Ming Fai, Ahmed Hussein Birima, Ahmed El-Shafie

**Affiliations:** 1 Department of Civil Engineering, Faculty of Engineering, Universiti Malaya (UM), 50603 Kuala Lumpur, Malaysia; 2 Department of Civil Engineering, College of Engineering, Universiti Tenaga Nasional (UNITEN), 43000 Kajang, Selangor Darul Ehsan Malaysia; 3 Department of Geography and Planning, University of Liverpool, Liverpool, L69 3BX UK; 4 Institute for Energy Infrastructure (IEI), Universiti Tenaga Nasional (UNITEN), 43000 Kajang, Selangor Darul Ehsan Malaysia; 5 Discipline of Civil Engineering, School of Engineering, Monash University Malaysia, Jalan Lagoon Selatan, Bandar Sunway, 47500 Selangor, Malaysia; 6 Department of Civil Engineering, College of Engineering, Qassim University, Unaizah, Saudi Arabia; 7 National Water and Energy Center, United Arab Emirate University, P.O. Box. 15551, Al Ain, UAE

**Keywords:** Environmental sciences, Civil engineering

## Abstract

Solar energy serves as a great alternative to fossil fuels as they are clean and renewable energy. Accurate solar radiation (SR) prediction can substantially lower down the impact cost pertaining to the development of solar energy. Lately, many SR forecasting system has been developed such as support vector machine, autoregressive moving average and artificial neural network (ANN). This paper presents a comprehensive study on the meteorological data and types of backpropagation (BP) algorithms used to train and develop the best SR predicting ANN model. The meteorological data, which includes temperature, relative humidity and wind speed are collected from a meteorological station from Kuala Terrenganu, Malaysia. Three different BP algorithms are employed into training the model i.e., Levenberg–Marquardt, Scaled Conjugate Gradient and Bayesian Regularization (BR). This paper presents a comparison study to select the best combination of meteorological data and BP algorithm which can develop the ANN model with the best predictive ability. The findings from this study shows that temperature and relative humidity both have high correlation with SR whereas wind temperature has little influence over SR. The results also showed that BR algorithm trained ANN models with maximum R of 0.8113 and minimum RMSE of 0.2581, outperform other algorithm trained models, as indicated by the performance score of the respective models.

## Introduction

### Background

Solar radiation (SR) is the fundamental source of the Earth's energy^1^, providing almost 99.97% of the heat energy needed for various chemical and physical processes in the atmosphere, ocean, land, and other water bodies^2^. Also, SR is the source of energy for the earth’s climate system^3^. According to Yadav and Chandel^4^, global solar radiation is considered as the most essential parameter in meteorology, renewable energy and solar energy conversion applications, especially for the sizing of standalone photovoltaic systems. Besides, SR prediction can also improve the planning and operation of photovoltaic systems and yield many economic advantages for electric utilities. Although fossil fuels can produce a large amount of energy, they are causing a lot of pollution at the same time. Moreover, fossil fuels are non-renewable, so they are bound to deplete in the near future. On the other hand, solar energy serves as a great alternative to fossil fuels as they are clean and renewable energy^5,6^, thus helping in reducing carbon emissions^7,8^. Many countries with great technology advancement have already taken the initiative to develop technologies and machines that could harness energy from the sun.

In the present day, solar energy, being a promising alternative energy source, has been greatly applied into our daily life^9^, such as solar-powered transportation, solar lighting, wearable solar techs e.g. cell phone, rechargeable flashlights, solar heating etc. Hence, it is very important that we are able to quantify solar radiation and predict how much is the sun emitting the radiation at a daily basis. Yacef, et al.^10^suggested that “one of the forecasting approaches being followed in recent times is the artificial intelligent technique to predict the solar radiation”. Fadare^11^ developed an ANN based model for prediction of solar energy potential in Nigeria. The outcome shows that the correlation coefficient between the ANN predictions and measured data exceeded 90%, thereby projecting a superior consistence of the model for assessment of solar radiation for locations in Nigeria. An ANN model to predict the daily global solar radiation in China was developed by Xiang, et al.^12^, which exhibited that the ANN model has higher accuracy as compared to other regression models.

An artificial neural network, which works similar to the human nervous system^13,14^, consists of an input layer of neurons (or nodes, units), one or two or even three hidden layers of neurons, and a final layer of output neurons^15–17^. ANNs have self-learning capabilities that enable them to produce better results as more data becomes available. ANNs are effective to simulate non-linear systems^18^. Hidden patterns, which could be independent of any mathematical models, can be found from the training data sets. If the same or similar patterns are met, ANNs come up with a result with minimum MSE. ANN maps the input vector into corresponding output vector and it is only imperative and other values need not be known. This makes ANNs very useful to mimic non-linear relationships without the need of any already existing models.

Moreover, different backpropagation algorithms were also considered while developing the ANN model to study the suitability of each algorithm in relation to the type of data that were fed into the model. The three backpropagation algorithms used in this study each have distinctive characteristics, which would in turn cause the ANN model to reflect different results despite having the exact same inputs. The LM algorithm typically requires more memory but less time. Training automatically stops when generalization stops improving, as indicated by an increase in the mean square error of the validation samples. As for the BR algorithm, this algorithm typically requires more time, but can result in good generalization for difficult, small or noisy datasets. Training stops according to adaptive weight minimization (regularization). Lastly, the SCG algorithm requires less memory. Training automatically stops when generalization stops improving, as indicated by an increase in the mean square error of the validation samples.

In this research, the following 4 different ANN models with different combinations of meteorological parameters (mean temperature, mean relative humidity and mean wind speed) are developed, each with 3 different back propagation algorithms for solar radiation prediction:

- Model I have the combination of 24-h mean temperature (^o^C) and 24-h mean relative humidity (%);
- Model II has the combination of 24-h mean temperature and 24-h mean windspeed (m/s);
- Model III has the combination of 24-h mean relative humidity (%) and 24-h mean windspeed (m/s);
- Model IV has all three of the meteorological inputs above. All 4 models only have one output, which is global solar radiation (MJm^−2^).

Among the four models, the best ANN model along with the backpropagation algorithm which exhibits the best predictive ability is selected based on the minimum mean absolute error (MAE), minimum root means square error (RMSE) and maximum linear correlation coefficient (R).

### Literature review

Sözen, et al.^19^ conducted a study on the forecast of solar potential in Turkey using neural network approach. The main objective of this study is to put forward to solar energy potential in Turkey using ANNs with the following back propagation algorithms: scaled conjugate gradient (SCG), Pola–Ribiere conjugate gradient (CGP), and Levenberg–Marquardt (LM) learning algorithms and logistic sigmoid transfer function. The inputs and outputs are normalized in the range of −1 to 1 and the ANN models are developed under MATLAB environment. The results obtained in terms of maximum mean absolute percentage error (MAPE) and absolute fraction of variance (R^2^) were also compared with other classical regression models to predict solar radiation. The results of validation and comparative study indicate that ANN based prediction model has the advantage as compared to those classical regression models.

Kisi and Uncuoğlu^20^ carried out a study on the performances of three BP algorithms, namely the LM, CG and RB for stream flow forecasting and determination of lateral stress in cohesionless soils. The study results showed that despite LM being the fastest and best performed algorithm (short training time and fast convergence speed) as compared to others in the training dataset, the RB algorithm was in fact the better algorithm in terms of accuracy for the testing dataset.

Following in the year of 2009, a study on the modelling of solar energy potential in Nigeria using ANN model by Fadare^11^ was carried out. In this study, standard multi-layered, feed-forward, back-propagation neural networks with different architecture were designed using the neural toolbox for MATLAB. The data used to train and validate the model were the geographical and meteorological data of 195 cities in Nigeria obtained from the NASA geo-satellite database. The results from this study showed that the correlation coefficients between the ANN predictions and the actual mean monthly global solar radiation were over 90%, thus indicating a high reliability of the model for evaluation of solar radiation. A graphical user interface (GUI) was also developed for the application of the model.

In research carried out by Xinxing, et al.^21^, they have categorized BP algorithm into 6 classes as adaptive momentum, self-adaptive learning rate, resilient backpropagation, conjugate gradient, quasi-newton, bayesian regularization. In this study, the performance of these algorithms is being evaluated in terms of their predictive ability, convergence speed and training duration based on an electricity load forecasting model. From this study, it is found that BR algorithms have a fairly low MAPE at 3.5% as compared to other training algorithms. However, this high performance maybe due to its heavy processing load, hence slower training time. Recommendations have been made where the processing ability is limited, resilient backpropagation or conjugate gradient may be employed to reduce the training duration and achieve a rather accurate result.

Mishra, et al.^22^ has carried out a study on the analysis of LM and SCG training algorithm using a MLP based ANN to estimate channel equalizers. The performance of the algorithms is evaluated based on least square (LS) and minimum mean square error (MMSE). From the study results, the predictive ability and training speed of both algorithms are analogous. However, in the context of MSE against Epoch graph, the LM does have better accuracy compared to SCG. This is due to a relatively smaller dataset and hence the LM outperformed SCG algorithm on a simple MLP structure.

Subsequently, in the year of 2016, a more detailed study on the prediction of solar radiation for solar systems by using ANN models with different back propagation algorithms by Premalatha and Valan Arasu^23^ further proved the ability of ANN models to predict solar radiation to a certain accuracy. In this research, two ANN models with four different algorithms are considered. The ANN models are evaluate based on the minimum mean absolute error (MAE) and root mean square error (RMSE) and maximum linear correlation coefficient (R) of their respective results. The objective of this study is to compare the 4 back propagation algorithms: gradient descent (GD), Levenberg–Marquardt (LM), resilient propagation (RP) and scaled conjugate gradient (SCG). The input parameters used in this study are latitude, longitude, altitude, year, month, mean ambient air temperature, mean station level pressure, mean wind speed and mean relative humidity. The output is the monthly average global solar radiation. The results show that the ANN model with the LM algorithm achieved minimum values of MAE and RMSE. It is also shown that the LM algorithm is able to converge well within a shorter period of time among the four algorithms used to provide an accurate solution with minimum error.

In the same year, Kayri^24^ conducted a study on the predictive ability of Bayesian Regularization and Levenberg–Marquardt algorithms in ANN based on a comparative empirical study on social data. The ANN model was tested with 1 to 5 neuron architectures respectively through MATLAB. From the results, it is concluded that the BR algorithm has a better performance compared to LM due to a higher correlation coefficient and lower SSE in terms of its predictive ability. Nevertheless, similar to the results from the study carried out by Kisi and Uncuoğlu^20^, the LM algorithm once again proved to be the algorithm with the fastest convergence due to a low MSE, it was still outperformed by the BR in terms of accuracy and predictive ability. Similarly, Okut, et al.^25^ also carried out an investigation on the predictive performance of BR and SCG algorithms. In their study, it is found that BRANN had a better performance but not significantly so.

Ghazvinian, et al.^26^ attempted to predict solar radiation by developing an integrated support vector regression and an improved particle swarm optimization-based model. A new prediction model for solar radiation based on support vector regression (SVR) is developed behind an improved particle swarm optimization (IPSO) algorithm. Different prediction models such as the M5 tree model (M5T), genetic programming (GP) and SVR integrated with different optimization algorithms e.g. SVR-PSO, SVR-IPSO, Genetic Algorithm (SVR-GA), FireFly Algorithm (SVR-FFA) and the multivariate adaptive regression (MARS) model were tested along with different input parameters. This study showed that the SVR-IPSO model is superior as compared to other presented models. The performance of the model can be further enhanced by adding other input variables that directly influence solar radiation.

Artificial neural networks (ANNs) are one of the most essential components of soft computing. They are used to replicate the functioning of the human brain and to analyse and process data. The ability of ANNs to self-learn allows them to calculate accurate responses to problems that are difficult to solve using traditional analytical methods. It can comprehend, ask, and learn without having to be reprogrammed, grasp missing data, be easily preserved, have high accuracy, be implemented on parallel hardware, and respond to nonlinear complicated models without imposing any limitations or assumptions on the incoming data. Because of their resilience and efficacy, neural network-based algorithms and stochastic methods have recently received a lot of interest in the fields of computer science, engineering, and ANN. The ANN has been widely used in different research areas and help solving complicated problems. In this context, through a Bayesian Regularization approach based on neural networks, physical parameters such as thermal relaxation parameter, prandtl number, fluid suction/injection, and stretching/shrinking sheet have been successfully computed as reported in^27^. In addition^28^, concluded that by varying surface thickness using trained Artificial Neural Networks and the Levenberg–Marquardt Back-propagation (ANNLMB) procedure, the strength of Back-propagated Intelligent Networks (BINs) is manipulated and showed outstanding performance for numerical investigations of randomness attributes in magnetohydrodynamics (MHD) nanofluidic flow model. Different dimension of ANN application has been developed to the second kind of Three-point singular boundary value problems (TPS-BVPs) by^29^. In this study, several enhancements to the ANN has been proposed utilizing different optimization techniques and algorithms to achieve better results and showed that the ANN modelling approach could solve such complex application. Furthermore, for addressing the HIV infection model of CD4 + T cells^30^, developed an integrated intelligent computing framework that used a layered structure of neural network with diverse neurons and their optimization with efficacy of global search using genetic algorithms. The study showed that the proposed ANN modelling approach is robust, trustworthy and convergent^31^ showed that the ANN could be successfully developed and implemented to solve the third-order nonlinear multiple singular systems represented with Emden–Fowler differential equation (EFDE).

## Materials and methods

In this study, the data provided are the meteorological data of Kuala Terengganu, Malaysia obtained from the Malaysian Meteorological Department. The data collected are the 24-h mean temperature, 24-h mean relative humidity, 24-h mean wind speed and global radiation from year 1985 to 2012. Besides, the latitude, longitude and elevation of the Kuala Terengganu meteorological station, covering the largest city in the area^32^, were also given at 5° 23′ N, 103° 06′ E and 5.2 m respectively. The meteorological data collected are solely based on one meteorological station so this may pose a problem of the data being less diversified as the climatic condition is pretty constant. Malaysia is located near to the equatorial line, hence the tropical rainforest climate with high rate of rainfall^33^ and overall high temperature throughout the entire year^34^ is observed at the location of our studies. Besides, the meteorological data at night may also be captured by the meteorological station, hence resulting at the zero values of global radiation. These extreme values may reduce the effectiveness of the learning ability of the ANN model, thus reducing its accuracy to predict solar radiation.

**Figure 1:**
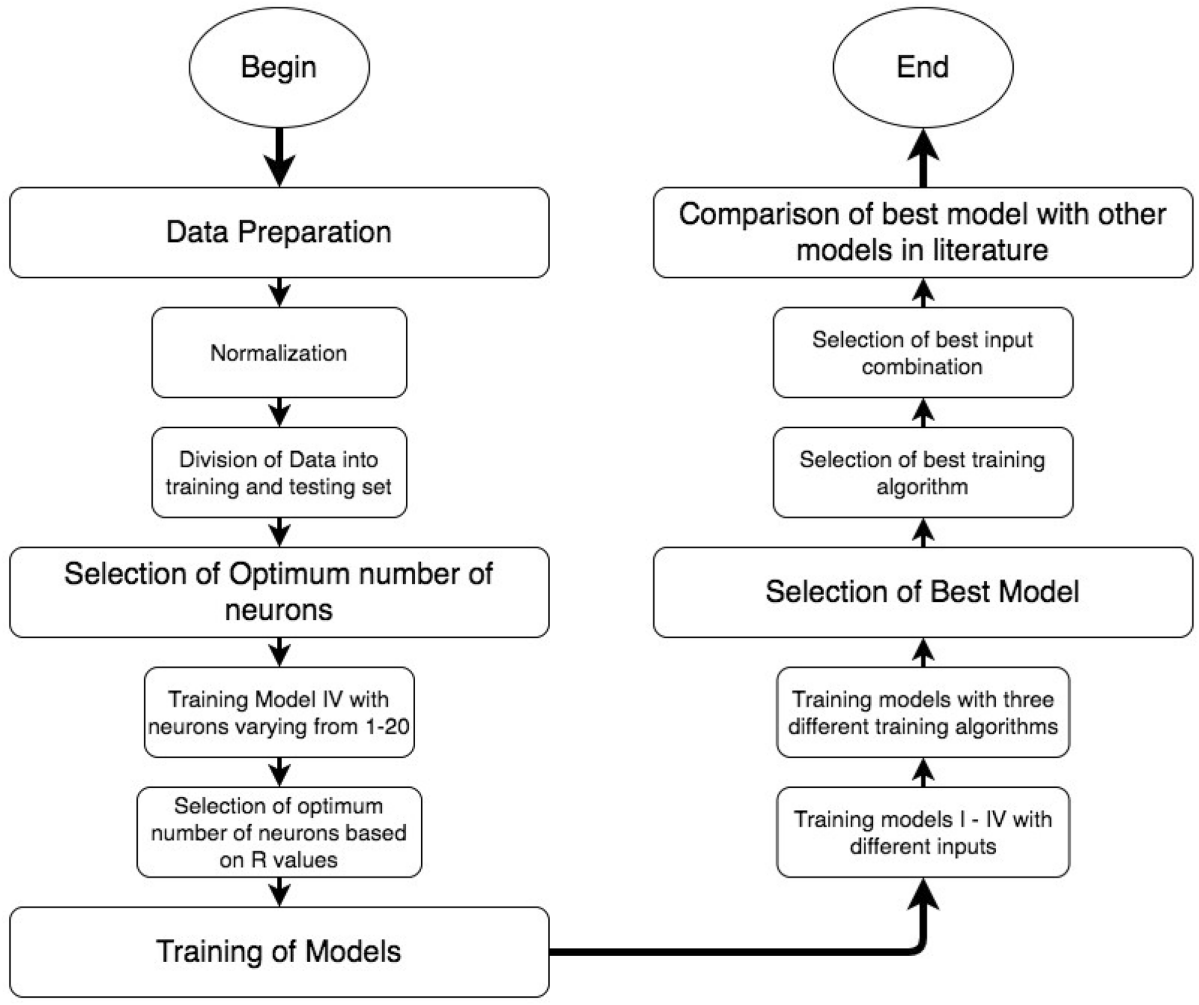
Flow chart of methodology.

The methodology of the current study follows the steps represented in Fig. 1. The obtained data is processed accordingly to prepare for ANN models training. Based on this data optimum neurons are selected which provides better training accuracy. The processed data and the selected optimum neurons are then used to train the intended models. Best models are selected using statistical analysis and are then compared with different model developed in literature.

### Preparation of data

Before employing the data to create and train the ANN model, the data have to go through normalization. Data normalization is a very common technique that is applied to prepare the data for machine learning. The objective of normalization is to alter the numeric values in the dataset to use a common scale, without distorting differences in the ranges of values or losing information. By normalizing the data, we are able to create new values from within the data that maintain the general distribution and ratios in the source data, while keeping values within a scale applied across all numeric columns used in the model. The meteorological data (inputs) are normalized by transforming them into values within the range of −1 and 1 using basic coding in MATLAB. Besides, the GSR has also been put through a log transformation to ensure the data is not too skewed and approximate to normality. The formula of normalization and log transformation is also shown as below:

1 $${X}_{N}= \frac{X-{X}_{min}}{{X}_{max}-{X}_{min}}$$

2 $${Y}_{T}=\mathrm{log}\left(1+Y\right)$$

where $X$ is meteorological data (temperature, relative humidity, windspeed), ${X}_{min}$ is minimum value of all available meteorological data, ${X}_{max}$ is maximum value of all available meteorological data, ${X}_{N}$= normalized meteorological data, Y_T_ = log transformed output (solar radiation) and Y = actual output. A total of 8431 samples of meteorological data from Kuala Terengganu are randomly divided according to the ratio; training = 70%, validation = 15% and testing = 15%. This ratio is maintained throughout the development of ANN for all four models. After this, number of hidden neurons is set at 15 as we have determined in Sect. 2.2.

### Selection of optimum number of hidden neurons

Determining the optimum number of hidden neurons in the hidden layer can be a very complicated process. Having the optimum number of hidden neurons is able to ensure a great accuracy of the ANN model and achieve a minimum possible error in the output. In order to select the optimum number of hidden neurons in the hidden layer, model IV was developed by increasing the number of neurons one by one until it converged into the smallest mean squared error. Since the Levenberg–Marquardt algorithm is the one that produces the highest R value (in the range of 0.7 to 0.8) among the four algorithms, the LM algorithm is used in the selection of the optimum number of hidden neurons.

As shown in Table 1 below, model IV is trained several times using different number of hidden neurons and the R values for both the training and testing is slowly increasing as the number of neurons is increased. When the hidden neuron is increased until 15, a maximum R value for both the training data and testing data is reached (0.8024 for training and 0.8231 for testing). This means that the predicted values show great correlation with the actual values and they are consistent with each other. When the number of hidden neurons is further increased up until 20, the R values are decreasing. Hence, the optimum number of hidden neurons in the hidden layer is determined to be 15 and shall be used in our present work.

**Table 1:** Selection of Optimum Number of Neurons based on R.

No. of neuron	R training	R testing
1	0.7408	0.7518
2	0.7507	0.7317
3	0.7985	0.7589
4	0.8026	0.7764
5	0.7997	0.7999
6	0.7926	0.8059
7	0.8029	0.7932
8	0.8053	0.7833
9	0.7946	0.8096
10	0.8043	0.8147
11	0.7995	0.8007
12	0.8102	0.8002
13	0.8077	0.7958
14	0.8012	0.8229
**15**	**0.8024**	**0.8231**
16	0.8059	0.7992
17	0.8105	0.7886
18	0.8101	0.7715
19	0.8113	0.802
20	0.8088	0.784

Significant values are in bold.

### Development of ANN model

**Figure 2:**
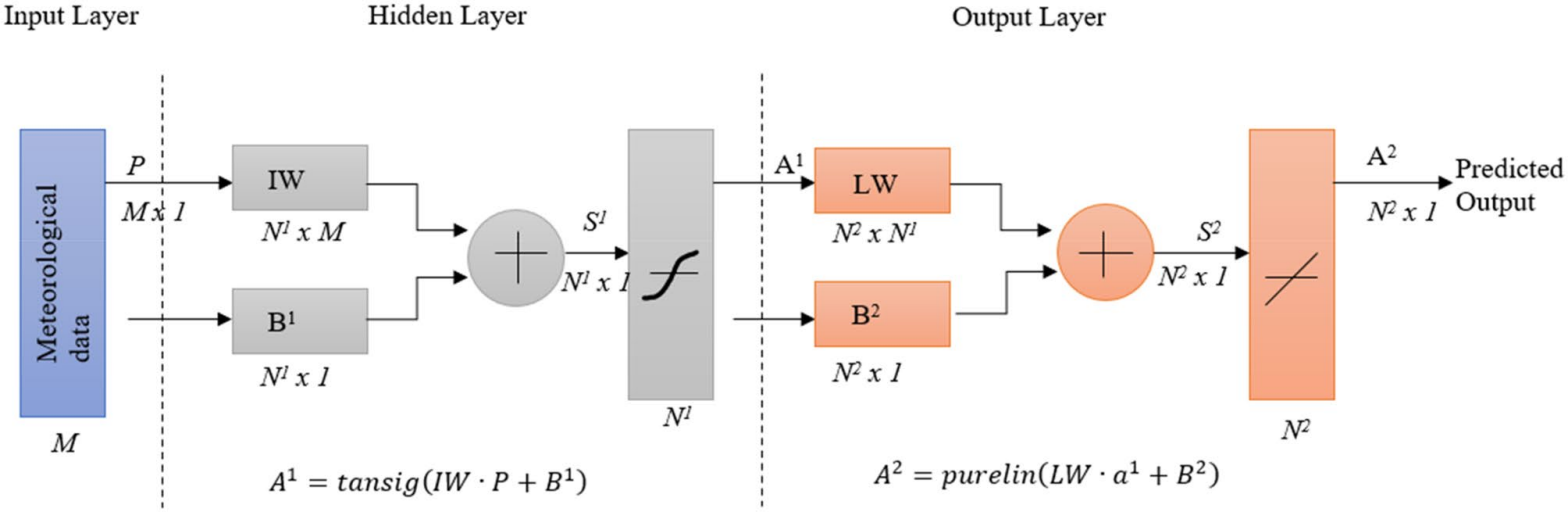
Architecture of ANN.

The architecture (Fig. 2) above demonstrate how does MATLAB train the network using the input data through a series of transfer and activate function, and subsequently being able to predict the output with minimum error. As seen from the figure above, the architecture of an ANN is made up of a series of individual component, which, in fact are represented in matrix form. The network above can be used as a general function approximator to approximate any function with a finite number of discontinuities.

The accuracy of the approximation depends on the number of neurons in the hidden layer. A few key parameters to take note in the architecture above is that *N*^*1*^ represents the number of neurons in the input layer while *M* represents the number of elements in the input layer. The IW (input weight) matrix is a *N*^*1*^* x M* matrix whereas B^1^ (bias vector) is a *N*^*1*^ length matrix. This is similar for the components in the output layer, only to be differentiated by the superscript 1 & 2 beside the matrix, where 1 would mean the matrix is associated with the input layer and 2 means that the matrix is associated with the output layer. A^1^ is the transformed matrix from the hidden layer that has undergone transformation through the tan-sig (tan-sigmoid) function. The tan-sig transfer function is a function that takes a matrix of net input vectors, *S*^*1*^ and returns the matrix A^1^ in the range of [−1, 1]. Similarly, A^2^ is a transformed matrix from the purelin (linear) function. Purelin transfer function works similarly as the tan-sig function, except for that it takes the input to transform it and return it linearly in the range of [−1, 1]. Hence, the ANN model will be train with three different backpropagation algorithm which is LM, SCG and BR to reduce the error rates and make more reliable model with increasing its generalization.

### Statistical analysis

The existing result generated from the model is not sufficient enough to evaluate the performance of ANN which is MSE and R, so generating a script is a must to strengthen the evaluation of performance by calculating the RMSE, MAE and NSE statistical analysis. The formula of RMSE, MAE and NSE are as below:

3 $$RMSE=\sqrt{\frac{1}{N}\sum_{i=1}^{N}{({X}_{i}-{Y}_{i})}^{2}}$$

4 $$MAE=\frac{1}{N}\sum_{i=1}^{N}\left|{X}_{i}-{Y}_{i}\right|$$

5 $$NSE=1-\frac{\sum {\left({Y}_{i}-{X}_{i}\right)}^{2}}{\sum {\left({X}_{i}-\overline{X }\right)}^{2}}$$

where ${X}_{i}$ = actual global solar radiation (MJm^−2^), ${Y}_{i}$ = predicted global solar radiation (MJm^−2^), N = total number of data, ${X}_{i}$ = actual global solar radiation (MJm^−2^), ${Y}_{i}$ = predicted global solar radiation (MJm^−2^), $\overline{X }$ = average of actual global solar radiation (MJm^−2^) and N = total number of data. In general, after running the script of the ANN, all the key information and data will be produced, such as the predicted output, the difference between the actual and predicted output, the performance of the model in terms of MSE. After running through the entire process for all four models with all three different backpropagation algorithms, all of the relevant parameters and key data are then recorded into an Excel sheet in order to carry out the appropriate evaluation on the models’ performance.

## Result and discussion

### Model I (temperature and relative humidity)

In Model I, two input parameters are considered here.

- 24-h mean relative humidity (%)
- 24-h mean wind speed (m/s)

The architecture of the neural network consisted of above-mentioned two input parameters, one hidden layer with 15 nodes in it and one output layer.

#### Levenberg–Marquardt

The results of Model I trained using the LM backpropagation algorithm is shown in Table 2.

**Table 2:** Evaluation using LM backpropagation.

Performance (MSE)	0.0848
MAE	0.203
RMSE	0.2913
R (training)	0.7451
R (testing)	0.7566
NSE	0.5682

**Figure 3:**
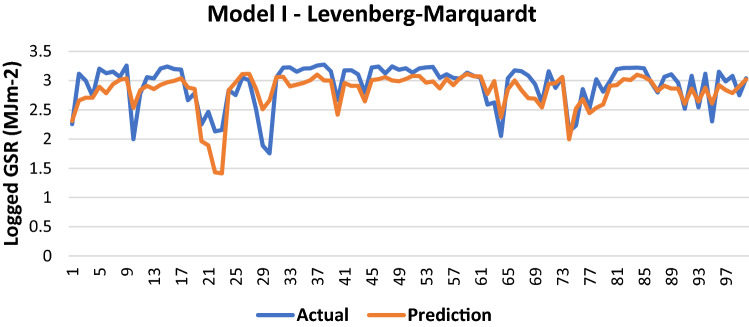
Graph of Actual and Predicted GSR (Model I—LM).

100 sets of actual and predicted GSR are randomly selected and plotted to aid in the visualization of the correlation between the actual values and the predicted values by the ANN model (Fig. 3).

#### Scaled conjugate gradient

The results of Model I trained using the SCG backpropagation algorithm is shown in Table 3.

**Table 3:** Evaluation using SCG backpropagation.

Performance (MSE)	0.0872
MAE	0.2073
RMSE	0.2953
R (training)	0.7366
R (testing)	0.7368
NSE	0.5556

**Figure 4:**
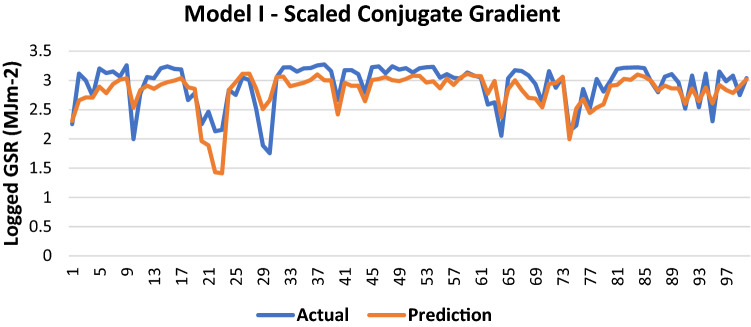
Graph of Actual and Predicted GSR (Model I—SCG).

100 sets of actual and predicted GSR are randomly selected and plotted to aid in the visualization of the correlation between the actual values and the predicted values by the ANN model (Fig. 4).

#### Bayesian regularization

The results of Model I trained using the BR backpropagation algorithm is shown in Table 4:

**Table 4:** Evaluation using BR backpropagation.

performance (MSE)	0.0872
MAE	0.2073
RMSE	0.2953
R (training)	0.7366
R (testing)	0.7368
NSE	0.5770

**Figure 5:**
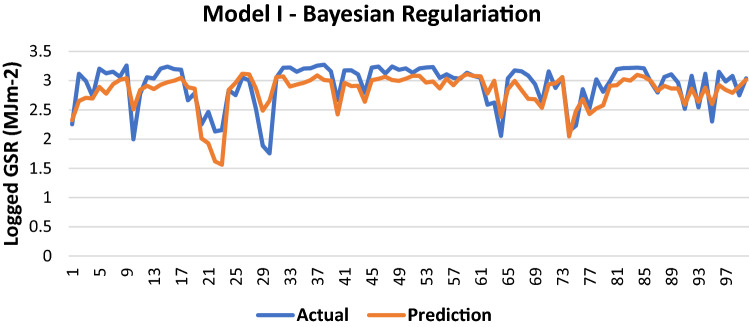
Graph of Actual and Predicted GSR (Model I—BR).

100 sets of actual and predicted GSR are randomly selected and plotted to aid in the visualization of the correlation between the actual values and the predicted values by the ANN model (Fig. 5).

### Model II (temperature and windspeed)

In Model II, two input parameters are considered here:

- 24-h mean temperature (^o^C)
- 24-h mean wind speed (m/s)

The architecture of the neural network consisted of above-mentioned two input parameters, one hidden layer with 15 nodes in it and one output layer.

#### Levenberg–Marquardt

The results of Model II trained using the LM backpropagation algorithm is shown in Table 5:

**Table 5:** Evaluation using LM backpropagation.

Performance (MSE)	0.0948
MAE	0.2104
RMSE	0.3078
R (training)	0.70791
R (testing)	0.71415
NSE	0.5145

**Figure 6:**
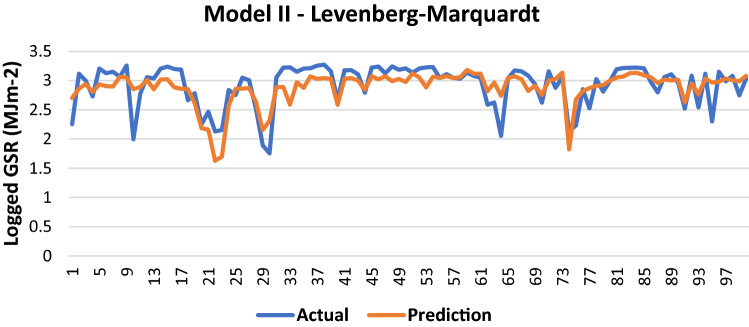
Graph of Actual and Predicted GSR (Model II—LM).

100 sets of actual and predicted GSR are randomly selected and plotted to aid in the visualization of the correlation between the actual values and the predicted values by the ANN model (Fig. 6).

#### Scaled conjugate gradient

The results of Model II trained using the SCG backpropagation algorithm is shown in Table 6.

**Table 6:** Evaluation using SCG backpropagation.

Performance (MSE)	0.1132
MAE	0.2282
RMSE	0.3364
R (training)	0.63468
R (testing)	0.64402
NSE	0.4165

**Figure 7:**
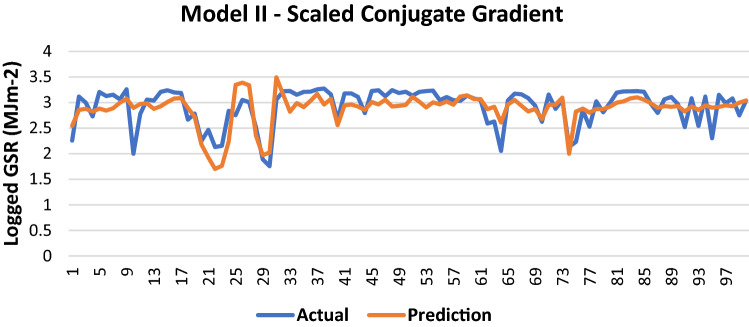
Graph of Actual and Predicted GSR (Model II—SCG).

100 sets of actual and predicted GSR are randomly selected and plotted to aid in the visualization of the correlation between the actual values and the predicted values by the ANN model (Fig. 7).

#### Bayesian regularization

The results of Model II trained using the BR backpropagation algorithm is shown in Table 7.

**Table 7:** Evaluation using BR backpropagation.

Performance (MSE)	0.0939
MAE	0.2094
RMSE	0.3065
R (training)	0.70673
R (testing)	0.74377
NSE	0.5192

**Figure 8:**
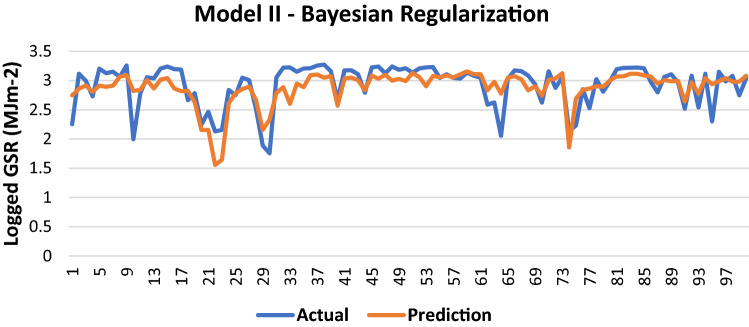
Graph of Actual and Predicted GSR (Model II—BR).

100 sets of actual and predicted GSR are randomly selected and plotted to aid in the visualization of the correlation between the actual values and the predicted values by the ANN model (Fig. 8).

### Model III (windspeed and relative humidity)

In Model III, two input parameters are considered here:

- 24-h mean temperature (^o^C)
- 24-h mean relative humidity (%)

The architecture of the neural network consisted of above-mentioned two input parameters, one hidden layer with 15 nodes in it and one output layer.

#### Levenberg–Marquardt

The results of Model III trained using the LM backpropagation algorithm is shown in Table 8.

**Table 8:** Evaluation using LM backpropagation.

Performance (MSE)	0.0746
MAE	0.1864
RMSE	0.2732
R (training)	0.78945
R (testing)	0.76435
NSE	0.6222

**Figure 9:**
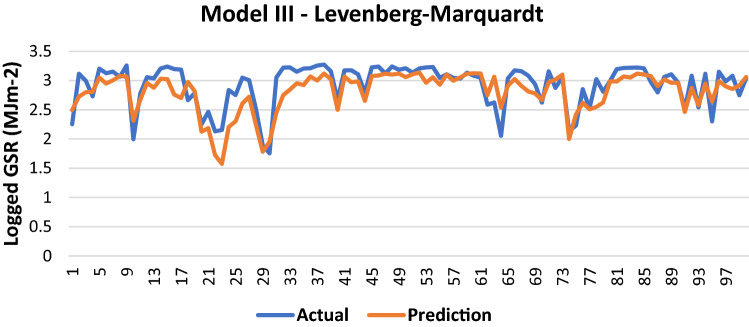
Graph of Actual and Predicted GSR (Model III—LM).

100 sets of actual and predicted GSR are randomly selected and plotted to aid in the visualization of the correlation between the actual values and the predicted values by the ANN model (Fig. 9).

#### Scaled conjugate gradient

The results of Model III trained using the SCG backpropagation algorithm is shown in Table 9:

**Table 9:** Evaluation using SCG backpropagation.

Performance (MSE)	0.0916
MAE	0.2096
RMSE	0.3027
R (training)	0.72979
R (testing)	0.70069
NSE	0.5306

**Figure 10:**
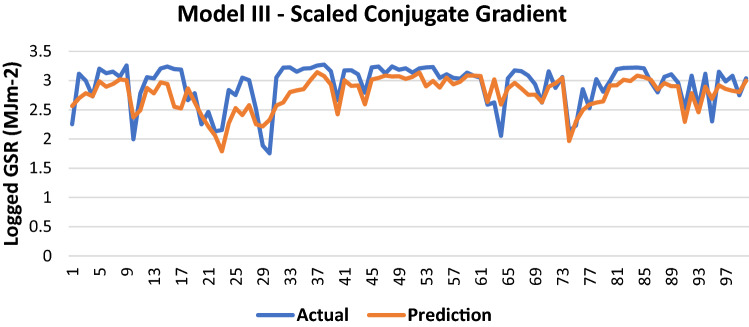
Graph of Actual and Predicted GSR (Model III—SCG).

100 sets of actual and predicted GSR are randomly selected and plotted to aid in the visualization of the correlation between the actual values and the predicted values by the ANN model (Fig. 10).

#### Bayesian regularization

The results of Model III trained using the BR backpropagation algorithm is shown in Table 10:

**Table 10:** Evaluation using BR backpropagation.

Performance (MSE)	0.0728
MAE	0.1872
RMSE	0.2698
R (training)	0.78724
R (testing)	0.78104
NSE	0.6313

**Figure 11:**
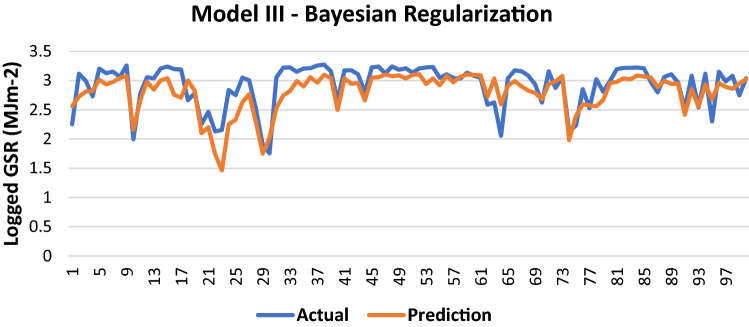
Graph of Actual and Predicted GSR (Model III—BR).

100 sets of actual and predicted GSR are randomly selected and plotted to aid in the visualization of the correlation between the actual values and the predicted values by the ANN model (Fig. 11).

### Model IV (temperature, relative humidity and windspeed)

In Model IV, three input parameters are considered here:

- 24-h mean temperature (^o^C)
- 24-h mean relative humidity (%)
- 24-h mean wind speed (m/s)

The architecture of the neural network consisted of above-mentioned three input parameters, one hidden layer with 15 nodes in it and one output layer.

#### Levenberg–Marquardt

The results of Model IV trained using the LM backpropagation algorithm is shown in Table 11:

**Table 11:** Evaluation using LM backpropagation.

Performance (MSE)	0.0687
MAE	0.183
RMSE	0.2621
R (training)	0.7955
R (testing)	0.8142
NSE	0.6536

**Figure 12:**
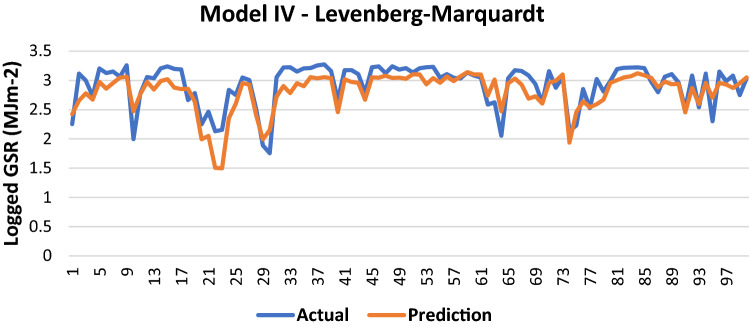
Graph of Actual and Predicted GSR (Model IV—LM).

100 sets of actual and predicted GSR are randomly selected and plotted to aid in the visualization of the correlation between the actual values and the predicted values by the ANN model (Fig. 12).

#### Scaled conjugate gradient

The results of Model IV trained using the SCG backpropagation algorithm is shown in Table 12.

**Table 12:** Evaluation using SCG backpropagation.

Performance (MSE)	0.0745
MAE	0.1897
RMSE	0.2729
R (training)	0.7807
R (testing)	0.79
NSE	0.6231

**Figure 13:**
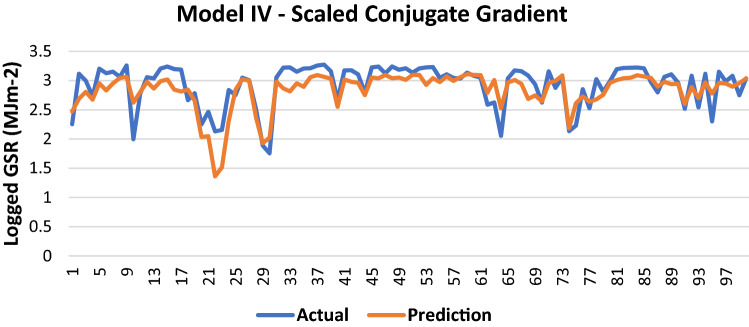
Graph of Actual and Predicted GSR (Model IV—SCG).

100 sets of actual and predicted GSR are randomly selected and plotted to aid in the visualization of the correlation between the actual values and the predicted values by the ANN model (Fig. 13).

#### Bayesian regularization

The results of Model IV trained using the BR backpropagation algorithm is shown in Table 13.

**Table 13:** Evaluation using BR backpropagation.

Performance (MSE)	0.0666
MAE	0.1789
RMSE	0.2581
R (training)	0.8059
R (testing)	0.8113
NSE	0.6654

**Figure 14:**
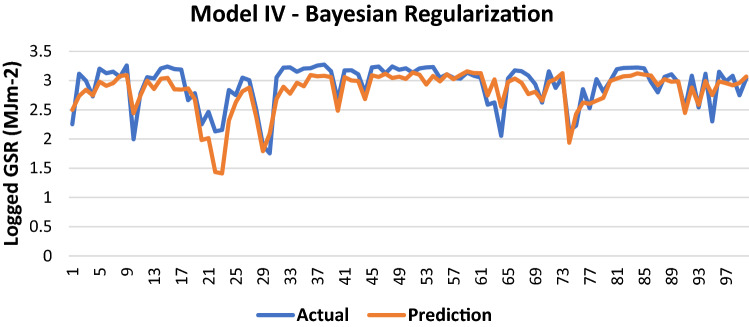
Graph of Actual and Predicted GSR (Model IV—BR).

100 sets of actual and predicted GSR are randomly selected and plotted to aid in the visualization of the correlation between the actual values and the predicted values by the ANN model (Fig. 14).

All the models developed above have same internal configuration (i.e. same number of hidden layer(1) and neurons(15)), but all the models differ in their input variables combination and training algorithms. The results of all the models consists of statistical values (i.e. MAE, RMSE, MSE, R and NSE) and a plot of actual and predicted GSR. All the plots look similar to each other, yet there is slight change in the plot which is reflected in its statistical values. Further comparison of the statistical values to produce the best training algorithm and best model with best combination of inputs are done in following sections.

### Selection of the best backpropagation algorithm

In order to determine the most suitable algorithm to be used in our ANN model for solar prediction, results tabulated in Table 14 will be used. In this case, a low MAE, RMSE, MSE values and a high R and NSE values will indicate that the algorithm used is effective in training the model and predicting SR.

**Table 14:** MAE, RMSE, MSE, R and NSE values of each model.

Model	Evaluation	Name of Algorithm		
		LM	SCG	BR
Model I	MAE	0.203	0.2073	*0.2015*
	RMSE	0.2913	0.2953	*0.2884*
	MSE	0.0848	0.0872	*0.0832*
	R	*0.7566*	0.7368	0.7565
	NSE	0.5682	0.5556	*0.5770*
Model II	MAE	0.2104	0.2282	*0.2094*
	RMSE	0.3078	0.3364	*0.3065*
	MSE	0.0948	0.1132	*0.0939*
	R	0.71415	0.64402	*0.74377*
	NSE	0.5145	0.4165	*0.5192*
Model III	MAE	*0.1864*	0.2096	0.1872
	RMSE	0.2732	0.3027	*0.2698*
	MSE	0.0746	0.0916	*0.0728*
	R	0.76435	0.70069	*0.78104*
	NSE	0.6222	0.5306	*0.6313*
Model IV	MAE	0.183	0.1897	*0.1789*
	RMSE	0.2621	0.2729	*0.2581*
	MSE	0.0687	0.0745	*0.0666*
	R	*0.8142*	0.79	0.8113
	NSE	0.6536	0.6231	*0.6654*

As seen from Table 14, the minimum MAE, RMSE, MSE values and maximum R and NSE values of each model are highlighted in Italics. The Bayesian Regularization algorithm has most of the best result, where it obtained a minimum value of 0.2015, 0.2094 and 0.1789 for MAE of Model I, Model II and Model IV respectively. Although the minimum MAE in Model III is LM’s 0.1864, but the difference is insignificant when compared to BR’s 0.1872, hence it is concluded that the BR algorithm has the best mean absolute error for all four models. Besides, BR algorithm also has the minimum RMSE values of 0.2884, 0.3065, 0.2698 and 0.2581 for Model I, Model II, Model III and Model IV respectively. By logic, BR algorithm will also obtain minimum MSE values for all four models since MSE is the square of RMSE. It can also be seen that BR algorithm has achieved maximum R values of 0.74377 and 0.78104 for Model II and Model III respectively. LM algorithm obtained the maximum R values for Model I and Model IV at 0.7566 and 0.8142, but the difference are again insignificant when compared to BR’s 0.7565 and 0.8113. Also, BR algorithm has achieved highest NSE value in all the four models i.e., 0.5770, 0.5192, 0.6313 and 0.6654 for model I, II, III and IV, respectively.

**Figure 15:**
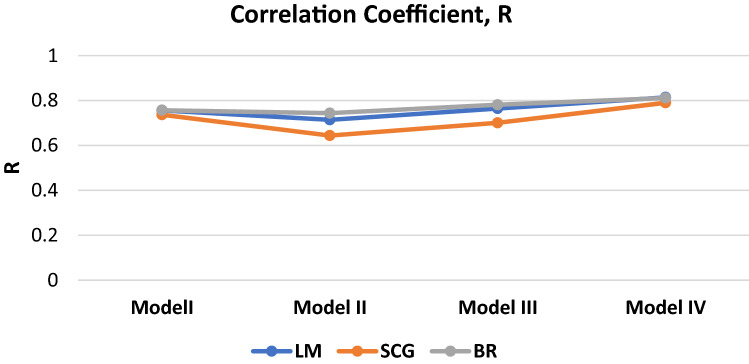
Graph of Correlation of Coefficient, R

The correlation coefficient is plotted and summarized in Fig. 15 for a clearer picture on the robustness of each BP algorithm.

Based on the results, it can be seen that the BR and LM trained ANN model are far superior when compared to the SCG trained model in terms of predictive ability and accuracy. This may be due to the SCG uses less memory in training the data as well as a faster training time (less average training iteration). However, when comparing the BR and LM algorithm, BR algorithm only has a slight edge over the LM algorithm as all of their key statistics are pretty close. This is due to the various advantages of the BR algorithm such as the robustness of the model, the probability distribution of network weights approach and optimization of the ANN architecture. This will help in overcoming overfitting issues due to a better data generalization ability. Hence, it can be said that the Bayesian Regularization BP algorithm is the best suited algorithm in our study to develop a SR predicting ANN model.

### Selection of the best ANN model

The comparison study above has proven BR algorithm to be the best suited algorithm to help train the ANN model. Now, a comparison between the models using the BR algorithm will be carried out to evaluate the meteorological data that are best used for SR prediction. The MAE, RMSE, R and NSE values of all four models are tabulated in Table 15 below.

**Table 15:** MAE, RMSE, MSE, R and NSE values of each model trained with BR.

Name of model	BR algorithm			
	MAE	RMSE	R	NSE
Model I	0.2015	0.2884	0.7565	0.5770
Model II	0.2094	0.3065	0.74377	0.5192
Model III	0.1872	0.2698	0.78104	0.6313
Model IV	0.1789	0.2581	0.8113	0.6654

As seen from Table 15 above, Model IV which has a combination of all the meteorological data as input is the best model as it has achieved minimum MAE, RMSE and maximum R and NSE at 0.1789, 0.2581, 0.8113 and 0.6654, respectively among the four models. All of these values appear to be better than the rest of the models which only used two of the three available meteorological data. Thus, it is concluded that if one wishes to achieve the greatest accuracy in SR prediction using ANN, mean temperature, mean relative humidity and mean wind speed should be used together as the input.

Now among the remaining 3 models, it can be seen that model III has the minimum MAE, RMSE and maximum R and NSE at 0.1872, 0.2698, 0.78104 and 0.6313, respectively as compared to Model I and Model II. The conclusion obtained from this comparison is that the combination of mean relative humidity and mean temperature will develop the ANN model with the best performance. We are also able to conclude that mean wind speed has little influence over solar radiation.

### Analysis of error

Based on the discussion above, it is found that BR algorithm is the best training algorithm and when employed in Model IV, the performance and accuracy of the predicted output is the best among the other models. This can be further justified by analysing the error of each models using mean absolute percentage error (MAPE). MAPE is a measure of prediction accuracy as a percentage and is commonly used to evaluate the accuracy of a forecast system, and in this case would be the ANN model. The formula of MAPE is given as below:

5 $$MAPE= \frac{1}{N}\sum_{i=1}^{N}\left|{(x}_{i}-{y}_{i}\right)/{x}_{i}| \times 100\mathrm{\%}$$

where ${x}_{i}$ = actual measured data, ${y}_{i}$ = predicted data and $N$ = total number of data.

Based on Table 16 above, it can be seen that the MAPE for all four models of different training algorithm are within the range of 10%—15%. The highest MAPE being 14.95%, which is from Model II’s SCG. This result is expected as we have stated in Sect. 3.5 that the SCG algorithm performs fairly poor when compared to the other algorithms, hence resulting in a greater error in the ANN models that were trained using SCG. In general, the MAPE of all the models trained with the BR algorithm are fairly low when compared to the LM and SCG trained models.

**Table 16:** MAPE of each model.

Name of Algorithm	Model I	Model II	Model III	Model IV
LM	12.39%	12.89%	11.79%	11.2%
SCG	12.07%	14.95%	14.41%	11.37%
BR	10.92%	12.51%	10.75%	10.64%

The lowest MAPE in the Table 16 is found to be 10.64%, which is the MAPE of Model IV trained using the BR algorithm. Again, this is an anticipated scenario that Model IV will be the ideal ANN for solar prediction as it has taken into consideration all three meteorological parameters and was trained by the most suitable algorithm. Hence, it is no surprise that it has recorded the lowest MAPE among all the models. A 10.64% of MAPE would mean that on average, the solar radiation prediction from ANN Model IV is off by 10.64%.

Nevertheless, it is difficult to set an arbitrary performance target to determine what is a good MAPE only based on comparison of different models conducted in the same study. Hence, the MAPE of other similar studies has been brought in to make a thorough comparison and evaluation.

As seen from Table 17, the MAPE of our present work (10.64%) is ranked at 3rd best when compared to similar studies of different location. The best MAPE is 6.78% (trained with ANN & SCG) and the worst MAPE is 19.1% (trained with ANN/MLFF). Hence, it can be determined that a MAPE of 10.64% is fairly acceptable, there is definitely room for improvement in terms of the predictive ability and robustness of the ANN model.

**Table 17:** Comparison of MAPE with similar studies.

Study	Station	MAPE (%)	Method/Algorithm
Mohandes, Rehman, and Halawani (1998)	Kwash (Saudi Arabia)	19.1	ANN/MLFF
Rehman and Mohandes (2008)	Abha (Saudi Arabia)	11.8	ANN/MLFF
Alawi and Hinai (1998)	Majees (North Oman)	7.30	ANN/MLFF
Sözen, Arcaklioǧlu, Özalp, and Caglar (2005)	Sirt (Turkey)	6.78	ANN/SCG
Present Study	Kuala Terrenganu (Malaysia)	10.64	ANN/BR

## Conclusion

Accurate SR prediction can substantially lower down the impact cost pertaining to the development of solar power. Due to the reliance on clear skies and inconsistency of atmospheric pressure and other meteorological parameters, SR prediction has become a rather challenging task which requires the help of artificial intelligence in order to correctly forecast the SR. In the present study, four ANN models have been developed to predict the solar radiation in Kuala Terrenganu, Malaysia. Each model is trained with three different BP algorithm i.e., Levenberg–Marquardt, Scaled Conjugate Gradient and Bayesian Regularization. The four models are each distinct in terms of the meteorological data that was used to train and develop the neural network. Model I have temperature and humidity; Model II has temperature and windspeed; Model III has humidity and windspeed; Model IV has temperature, humidity and windspeed. The study is carried out in such a way that the best suited training algorithm and combination of meteorological data for a SR predicting ANN model can be determined. From the findings of present study, it is determined that despite having almost similar performance scores (MSE, RMSE, MAE, R & NSE), the BR algorithm still outperformed the LM algorithm in terms of predictive ability as seen from the MAPE scores. The MAPE scores of Model I – Model IV trained by BR are 10.92%, 12.51%, 10.75% and 10.64% respectively whereas the MAPE scores of the same models trained by LM are 12.39%, 12.89%, 11.79% and 11.2% respectively. Besides, SCG trained models have the worst MAPE scores as some are even < 14%. The superior feature of the ANN model developed with BR algorithm is that it has a robustly built structure and better data generalization, which allows it to prevent overfitting issues. Hence, it is concluded that BR should be used in an ANN model for SR prediction.

Besides, the results also showed a rather high correlation between the measured SR and predicted SR when temperature and relative humidity are used as the meteorological input to train the model as compared to other combination of meteorological data which includes wind speed. This distinct can be seen from the MSE, RMSE, R and MAPE values between Model I, II and III. From this, it is concluded that temperature and relative humidity are closely related to SR whereas wind speed has little influence over it. A study by Yazdani, et al.^35^ also displayed similar results as their study results show that SR is directly proportional to atmospheric temperature while inversely proportional to the relative humidity. It is also found that wind speed does not affect solar radiation as much as the other two meteorological parameters. Nevertheless, when Model IV is developed with all three meteorological parameters as the input, it soon become the model with the best performance and highest accuracy among the four models. This also prove that the addition of wind speed parameter does enhance the learning ability of the ANN model, thus reducing the error (as seen from the MAPE score of Model IV being the lowest: 10.64%) in the predicted SR of Model IV. Finally, the results obtained does indeed prove that artificial neural network model can be used as a reliable forecasting system for solar radiation in certain location in Malaysia which shares the same climatic condition as Kuala Terrenganu. However, as ANNs are very sensitive to the initial parameters such as the input and output datasets, the performance and results might vary accordingly.

For future research works, it is recommended to train the ANN models with meteorological data collected from several different location. This would help in increasing the learning ability of the model as the training datasets will be more diversified, hence providing a better predictive ability for the model.

## Data availability

The data that support the findings of this study are available at Malaysian Meteorological Department.
